# Let Sleeping Patients Lie, avoiding unnecessary overnight vitals monitoring using a clinically based deep-learning model

**DOI:** 10.1038/s41746-020-00355-7

**Published:** 2020-11-13

**Authors:** Viktor Tóth, Marsha Meytlis, Douglas P. Barnaby, Kevin R. Bock, Michael I. Oppenheim, Yousef Al-Abed, Thomas McGinn, Karina W. Davidson, Lance B. Becker, Jamie S. Hirsch, Theodoros P. Zanos

**Affiliations:** 1 Institute of Bioelectronic Medicine, Feinstein Institutes for Medical Research, Northwell Health, Manhasset, NY USA; 2 Department of Information Services, Northwell Health, New Hyde Park, NY USA; 3 Donald and Barbara Zucker School of Medicine at Hofstra/Northwell, Hempstead, NY USA; 4 Institute of Health Innovations and Outcomes Research, Feinstein Institutes for Medical Research, Northwell Health, Manhasset, NY USA

**Keywords:** Health services, Computer science

## Abstract

Impaired sleep for hospital patients is an all too common reality. Sleep disruptions due to unnecessary overnight vital sign monitoring are associated with delirium, cognitive impairment, weakened immunity, hypertension, increased stress, and mortality. It is also one of the most common complaints of hospital patients while imposing additional burdens on healthcare providers. Previous efforts to forgo overnight vital sign measurements and improve patient sleep used providers’ subjective stability assessment or utilized an expanded, thus harder to retrieve, set of vitals and laboratory results to predict overnight clinical risk. Here, we present a model that incorporates past values of a small set of vital signs and predicts overnight stability for any given patient-night. Using data obtained from a multi-hospital health system between 2012 and 2019, a recurrent deep neural network was trained and evaluated using ~2.3 million admissions and 26 million vital sign assessments. The algorithm is agnostic to patient location, condition, and demographics, and relies only on sequences of five vital sign measurements, a calculated Modified Early Warning Score, and patient age. We achieved an area under the receiver operating characteristic curve of 0.966 (95% confidence interval [CI] 0.956–0.967) on the retrospective testing set, and 0.971 (95% CI 0.965–0.974) on the prospective set to predict overnight patient stability. The model enables safe avoidance of overnight monitoring for ~50% of patient-nights, while only misclassifying 2 out of 10,000 patient-nights as stable. Our approach is straightforward to deploy, only requires regularly obtained vital signs, and delivers easily actionable clinical predictions for a peaceful sleep in hospitals.

## Introduction

Poor sleep in hospitals is a common problem, with up to half of admitted patients experiencing insomnia^1,2^. Sleep disruptions are associated with undesirable effects ranging from delirium and cognitive impairment to weakened immunity, hypertension, elevated stress hormones and increased mortality^3,4^. Impaired sleep is also one of the most common complaints in hospitalized patients^1,3,5,6^, associated with significantly lower patient satisfaction^2^. The underlying causes of impaired sleep are multifactorial and include the effects of acute illness, pain, medications, and pre-existing conditions (e.g., sleep apnea), as well as environmental factors, such as noise, light, unfamiliar surroundings, and patient care activities^3,5^. This latter group of factors is among the most common causes of sleep disruption in hospitalized patients and maybe the most amenable to targeted initiatives^2,7^. Overnight vital sign (VS) monitoring, specifically, has been identified as an important cause of fragmented sleep whose utility has come under increased scrutiny^8^. Although no established evidence-based guidelines exist^9^, routine VS assessments commonly occur every four to five hours for medical and surgical patients regardless of patient acuity. Recent studies demonstrated fewer sleep interruptions and improved patient experience through targeted reductions in overnight VS measurements and medication administrations; however, determining which patients can forgo VS was left to subjective clinical assessment^10^.

While overnight VS measurements disrupt sleep, they are often indicated and necessary for high-risk and potentially unstable patients. Identifying these patients in a reliable and timely manner is an area of active investigation, with efforts focused on models that vary from single parameter tools to weighted early warning scores and advanced predictive models using machine learning techniques^11–14^. By contrast, relatively little work has been done to identify the low-risk cohort^15^ unlikely to benefit from such care that may, in fact, be harmed by these frequent assessments. Identifying this subset of patients has the potential to enhance recovery, improve patient sleep and satisfaction, and allow the redistribution of scarce resources (i.e., nurses, physicians) to higher-risk patients.

**Fig. 1:**
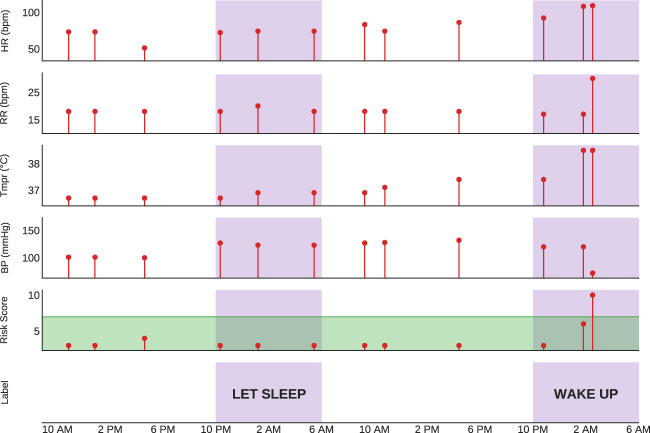
Example of the input sequence and predicted overnight stability of a hospital visit. The model takes a sequence of features as input, with each sample containing four vital signs plus a risk score (included in the figure) and other input variables, including an hour of the day, time since the previous sample, change in risk score, and variance of risk subscores (not shown). The green band signifies the normal range of risk score values and indicates low risk for clinical deterioration. Nights (purple band) are labelled according to the model prediction at 10 p.m. each night (“Let Sleep” or “Wake Up”). In the example provided, on the first night, the model predicted overnight stability and recommended that the patient sleep, whereas on the second night the model correctly predicted instability (i.e., elevated overnight risk score), thus recommending that the patient be woken for vital sign monitoring. Note that while in reality the model does not include measurements obtained during a predicted stable night, these are included in the figure for illustrative purposes. HR heart rate, RR respiratory rate, Tmpr temperature, BP systolic blood pressure, Risk score indicates Modified Early Warning Score (MEWS).

The goal of this project is to derive a tool that can reliably identify the subset of patients at extremely low risk for overnight deterioration. This project inverts the standard approach of identifying a high-risk cohort for closer monitoring and additional resources, and instead seeks to identify low-risk, stable patients who can safely avoid overnight VS assessments. By combining a deep recurrent neural network (RNN) for advanced predictive modeling with the clinical data (Fig. 1) generated by a multi-hospital health system, the derived tool enables the identification of low-risk inpatients and may improve outcomes by reducing overnight awakenings and enhancing sleep and recovery.

## Results

### Data description

**Fig. 2:**
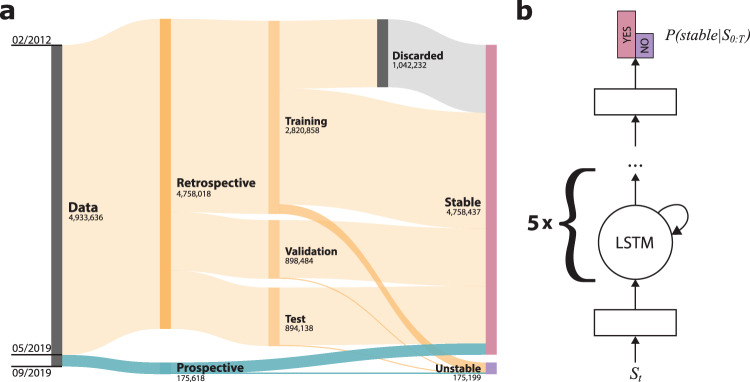
Data organization and model architecture. The complete de-identified retrospective and prospective data set contains 2,318,506 inpatient hospital admissions and 26,201,030 records of vital signs (VSs) between 2012 and 2019, yielding 4,933,636 VS input sequences used by our model (shown). The retrospective data set included all data through April 2019, while the prospective collection ranged from May to August 2019. Unrealistic, possibly mistyped VS values and records with any missing data after imputation were discarded. We split the retrospective data into training (70%), validation (15%), and test (15%) sets by patient visit, so the sequences drawn from one visit were only included in one of the groups. Some training data sequences with stable outcomes were discarded to balance the positive and negative cases (**a**). We used a deep recurrent neural network with two dense layers and five successive long short-term memory (LSTM) cells. After receiving a sequence of samples S0:T, the model predicts the probability of the patient becoming unstable during the night. We inserted a batch norm and dropout layer before the last fully connected layer (**b**).

The final data set obtained from a multi-hospital health system between 2012 and 2019 consisted of 2.13 million patient-visits (24.29 million VS measurements) in the retrospective cohort and 186,375 patient-visits (1.91 million VS measurements) in the prospective test set (Fig. 2a and Tables 1 and 2). We trained a deep RNN with long short-term memory (LSTM) cells to predict individual patient stability for any hospital night (Fig. 2b), using a sequence of prior VS records during the hospital stay of each patient. The algorithm ingests a parsimonious list of longitudinal features, including respiratory rate (RR), heart rate (HR), systolic blood pressure (BP), body temperature (Tmpr), patient age, and a calculated risk score (Modified Early Warning Score [MEWS]), and produces a nightly assessment of overnight stability.

**Table 1:** Data summary statistics.

	Retrospective	Prospective
Visits, *n*	2,132,131	186,375
Vitals, *n*	24,288,165	1,912,865
Age, mean (SD)	64.4 (19.0)	64.0 (18.8)
Gender (% female)	57	56.1
Heart rate (b.p.m.), mean (SD)	81.3 (16.6)	81.2 (16.8)
Respiratory rate (b.p.m.), mean (SD)	17.8 (2.0)	17.8 (2.0)
Systolic blood pressure (mmHg), mean (SD)	128.4 (22.6)	129.0 (23.0)
Temperature (°C), mean (SD)	36.8 (0.5)	36.8 (0.5)
Modified Early Warning Score, median (IQR)	3 (2)	3 (2)

**Table 2:** Outcome-based model input statistics.

	Stable	Potentially unstable
Age, mean (SD)	66.8 (17.8)	80.6 (12.8)
Heart rate (b.p.m.), mean (SD)	81.0 (14.9)	110.7 (25.0)
Respiratory rate (b.p.m.), mean (SD)	17.7 (1.5)	20.3 (4.5)
Systolic blood pressure (mmHg), mean (SD)	128.7 (21.2)	123.2 (32.0)
Temperature (°C), mean (SD)	36.8 (0.5)	37.3 (1.3)
Modified Early Warning Score, median (IQR)	3 (2)	7 (0)

An instance of a patient visit and associated VS trajectories is displayed in Fig. 1. As shown in the left portion of the figure, the combination of the patient’s VS and MEWS risk score led to a model prediction of “stable” during the first night. In this instance, the algorithm suggests that overnight VS assessment should be skipped and that the patient should be left sleeping. However, just prior to the second night in this example, the model accurately predicted a potentially unstable night, and thus recommended wakening the patient and checking VS, which uncovered an elevated MEWS of 10.

### Performance of the proposed model

**Fig. 3:**
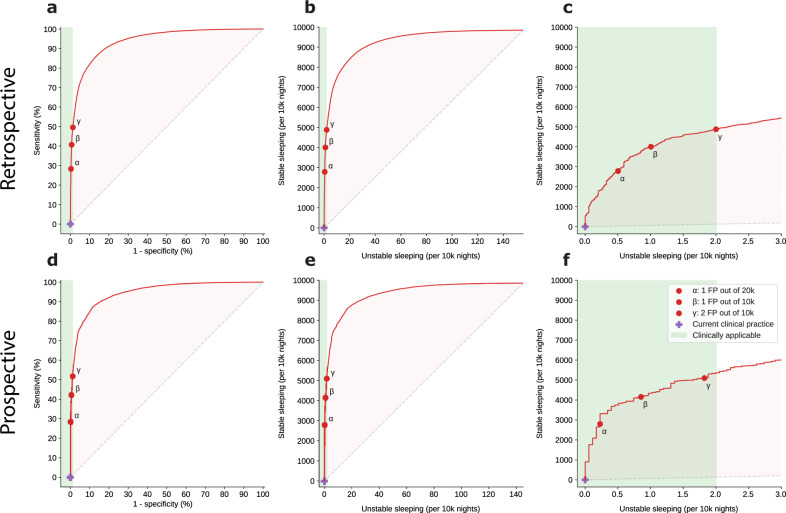
Model performance illustrated by receiver operating characteristic (ROC) curves and clinically renormalized variants. ROC curves for predicting overnight stability on the retrospective test (**a**) and prospective (**b**) datasets. The clinically renormalized variants for retrospective (**b**, **c**) and prospective (**e**, **f**) datasets show the balance between correctly letting stable patients sleep (*Y*-axis) and erroneously recommending sleep (predicted stable) to patients who ultimately had an elevated overnight risk score (*X*-axis), normalized to 10,000 patient-nights. The zoomed-in panels (**c**, **f**) highlight the clinically applicable range, which minimizes the number of false-positive predictions. The red points on all the curves (*α*, *β*, and *γ*) represent three different clinically applicable model thresholds, which were chosen according to the number of false positives they yielded for the retrospective test set. For example, threshold *γ*, the least conservative, maintains the number of unstable sleeping nights at the edge of acceptability, at 2 out of 10,000 patient-nights, while allowing approximately half of all patients to sleep safely (5000 out of 10,000 patient-nights). The blue cross on the bottom left of all panels indicates current practice, where all patients are woken for vital sign monitoring regardless of the risk level.

Using the proposed deep-learning predictive model, we achieved an area under the receiver operating characteristic (ROC) curve (AUC) of 0.966 (95% confidence interval [CI] 0.956–0.967) on the retrospective testing set, and 0.971 (95% CI 0.965–0.974) on the prospective set (Fig. 3). Following model training and ROC curve construction, we established three different confidence thresholds (Fig. 3, items *α*, *β*, and *γ*), out of which the least conservative, threshold *γ*, can avoid overnight VS for 50% of patient-nights, while only misclassifying as stable 2 out of 10,000 patient-nights. As shown in Fig. 3 (green portion of ROC curves), we established the clinically applicable region for this particular problem at a maximum of two false-positive predictions per 10,000 patient-nights (1.29% false-positive rate [patient-nights misclassified as stable divided by the total unstable patient-nights]), with the primary model threshold, *γ*, lying on this region’s edge. Thresholds *α* and *β* correspond to increasingly conservative models, with false-positive rates of 0.32% and 0.65%, respectively. The Mathew correlation coefficients are 0.082, 0.100, and 0.118, and the F1 scores are 0.473, 0.573, and 0.656 at the *α*, *β*, and *γ* confidence thresholds, respectively.

### Performance of a linear control model

To determine the benefit of using the proposed RNN architecture, we also evaluated a simple logistic regression model, receiving the same input variables as the proposed RNN, using the latest instance of VS measured right before the predicted patient-night rather than a sequence of VS. The logistic regression model achieved an AUC of 0.960 (95% CI 0.959–0.961) on the retrospective testing set and 0.964 (95% CI 0.962–0.965) on the prospective set (Supplementary Fig. 1).

### Falsely classified cases

**Fig. 4:**
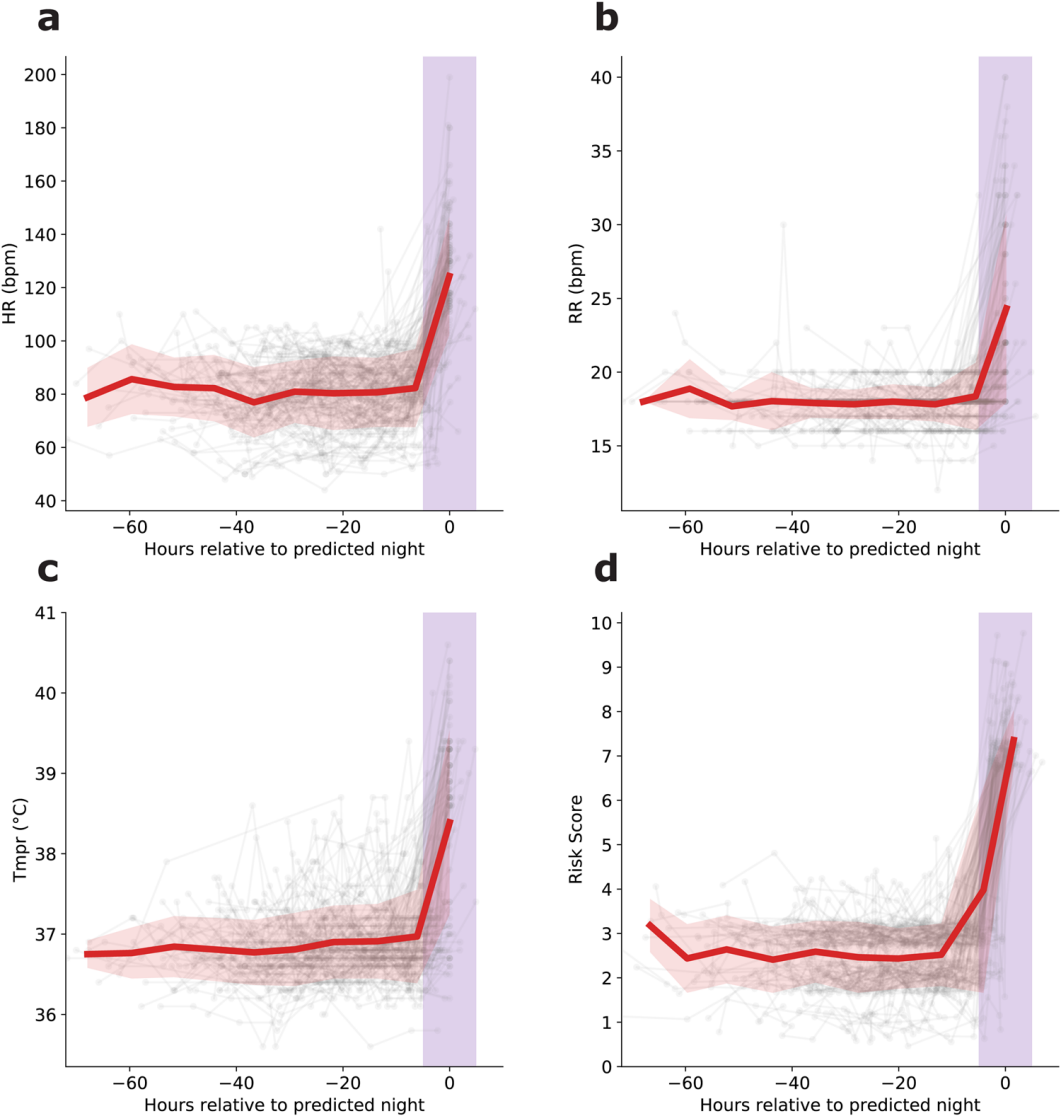
Trajectories of vital signs and risk scores preceding false-positive predictions. The vital sign and risk score trajectories of patients in the 72 h prior to erroneous predictions of overnight stability (false-positive). The vital signs and risk scores are largely stable with no suggestion of risk, but abruptly worsen during the overnight period. Red line represents mean trajectory; pink band covers two standard deviations; purple band demarcates the unstable night. Risk score indicates Modified Early Warning Score (MEWS), HR heart rate (b.p.m.), RR respiratory rate (b.p.m.), Tmpr temperature (°C).

Our proposed model misclassified 132 patient-nights as stable in our retrospective test data set. To understand the limitations of our model through the characteristics of these falsely classified cases, we inspected the preceding 72-h trajectories of risk scores and VS for the false-positive nights. The risk score, HR, RR, and Tmpr, for these cases stayed largely stable throughout the 3-day period, and abruptly increased during the falsely classified night (Fig. 4), underscoring the absence of any visible trend that could potentially inform a VS-based predictive model.

**Fig. 5:**
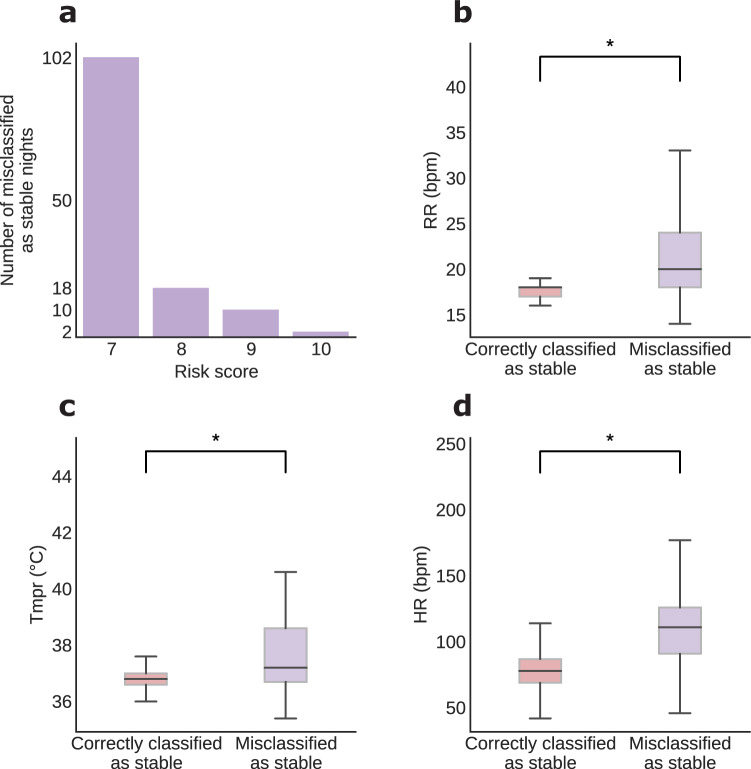
Differences in nighttime vital sign measurements between patients correctly and erroneously classified as stable in the retrospective test set. For the 132, erroneously classified as stable patient, 77% had a risk score of 7, which just met the threshold of potentially unstable, and in only 2 instances did the level reach up to 10 (**a**). Vital signs measured during stable (true positive) and potentially unstable (false positive) were compared, and all were found to be significantly different between the two groups, with respiratory rate (RR; **b**), temperature (Tmpr, **c**), and heart rate (HR, **d**) all higher in the misclassified as stable patients (*p* < 0.001 for all comparisons).

While the risk score and VS trends are not informative enough to guide the model, we examined the severity of the falsely classified cases, as well as the specific differences between cases where we correctly predicted stability and those misclassified as stable that ultimately were potentially unstable nights. In the vast majority of the 132 falsely classified cases (77%), the risk scores barely exceeded the chosen threshold of 7, with only 2 cases reaching a maximum risk score of 10 out of 15 (Fig. 5a). This distribution of risk scores implies that even in the rare cases of stability misclassification, most misclassified as stable cases correspond to marginally unstable patient-nights.

Moreover, HR, RR, and Tmpr were significantly higher in the incorrectly identified cases (Fig. 5b–d). More specifically, the RR of the patients during the misclassified nights (median 20.02 [interquartile range (IQR) 6]) were significantly higher than for the stable sleeping patients (median 18 [IQR 1]) (Fig. 5b; Kruskal–Wallis test, *p* < 0.01). Similarly, the HR (median 111 [IQR 35]) and Tmpr (median 37.2 [IQR 1.9]) during misclassified nights significantly exceeded those of patients sleeping stable (HR median 78 [IQR 18]; Tmpr median 36.8 [IQR = 0.4]) (Fig. 5c, d, respectively; Kruskal–Wallis test, *p* < 0.001 for all comparisons). In particular, the elevated overnight values of HR and RR in the misclassified as stable cases suggest that most patients would not be sleeping soundly, even with formal VS assessments skipped (86 cases with RR > 20 and 76 cases with HR > 120). Thus, a simple discreet visual inspection during routine nurse rounding or selective use of wearable devices could further identify most of these few misclassified as stable cases.

**Fig. 6:**
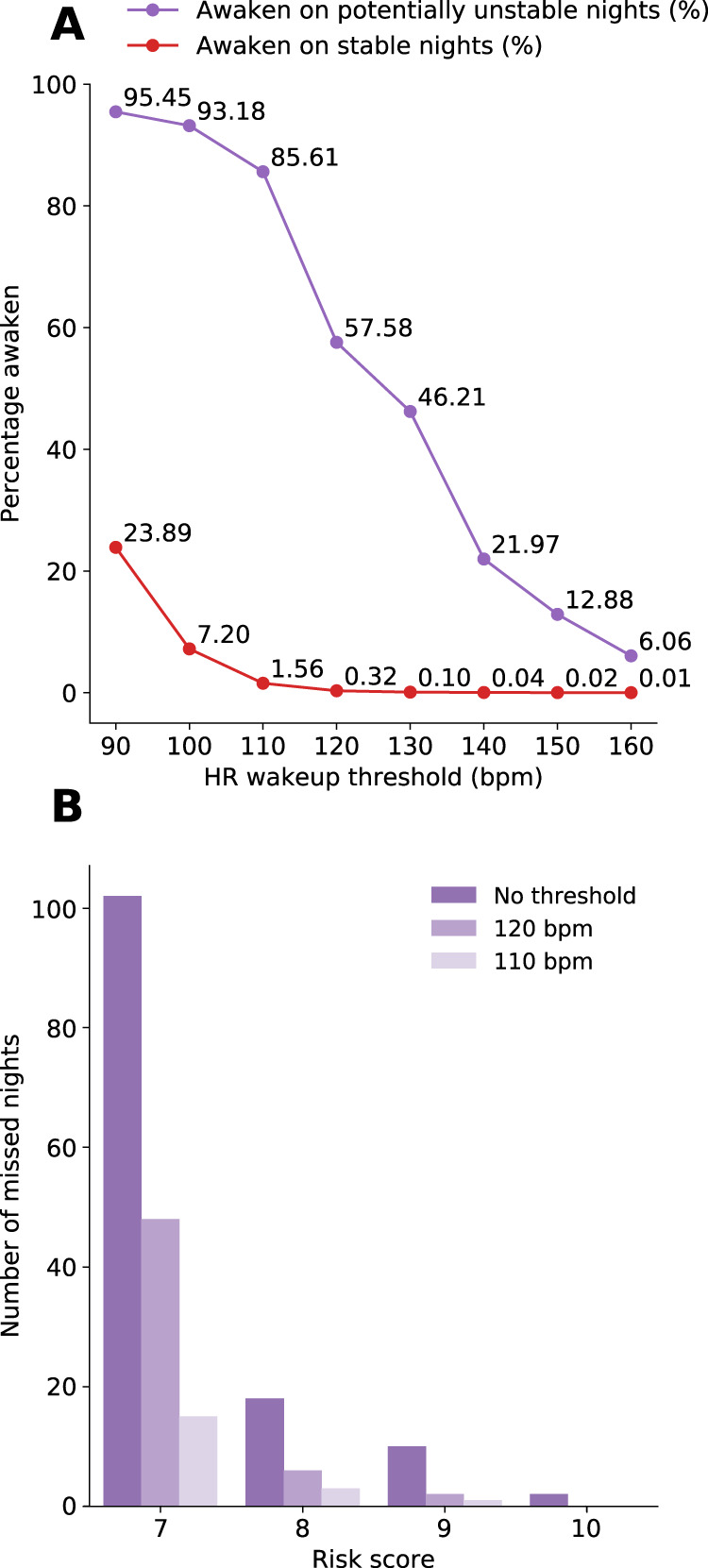
Detection of the instability of misclassified patients by overnight heart rate thresholds. Using only continuous heart rate monitors, and setting simple thresholds for alerting could facilitate patient recovery of erroneously classified patients who are potentially unstable. At various waking thresholds, most potentially unstable patients will be woken while some stable patients will also be woken (e.g., at the level of 100 b.p.m., 93.2% of potentially unstable patients and 7.2% of stable patients are additionally woken for assessment) (**a**). In the test set, 132 patients were misclassified as stable despite having a potentially unstable night. Using a threshold of 110 or 120 b.p.m., 113 (86%) and 76 (58%) potentially unstable sleepers, respectively, are identified, and the highest risk (risk score ≥10) are eliminated (**b**). b.p.m. Beats per minute.

While not individually predictive, HR and RR values could potentially be used as additional overnight discriminants to trigger a clinical assessment. Leveraging novel non-obstructive, continuous monitoring devices, patients erroneously predicted to be stable overnight could be woken for assessment based upon automatically captured and detected HR and RR thresholds. Figure 6 shows the tradeoff of using real-time overnight HR thresholds to assess sleeping patients previously classified as stable. Evaluating patients with HR exceeding 110 or 120 recovers 113 (86%) and 76 (58%) potentially unstable sleepers, respectively, while completely eliminating the cases of misclassified patient-nights with a risk score ≥10.

## Discussion

In this study, we successfully developed a generalizable deep-learning predictive model that takes a sequence of prior VS for a given patient and predicts the likelihood of overnight stability, in order to safely guide avoidance of overnight VS monitoring and unnecessary sleep disruption. The output of this model can salvage approximately half of patient-nights in a hospital or health system, avoiding unnecessary assessments while leaving patients undisturbed. These gains are achieved with extremely low risk, as our model misclassifies as stable only 2 out of 10,000 patient-nights. Moreover, the model is configured such that clinical teams can adjust the confidence threshold to implement a more stringent or risk-averse solution.

We evaluated a simple logistic regression model, receiving the same input variables as the proposed RNN, that uses the latest instance of VS measured right before the predicted patient-night rather than a sequence of VS. Although the linear model performed significantly worse on the task (random permutation test of AUC ROC scores, *p* < 0.05), saving 7% less patient-nights, we believe that such a model under data or computing power constraints can be employed with a high degree of success over the current clinical practice.

Our findings highlight the safety and accuracy of a machine learning-based solution to avoid unnecessary overnight VS monitoring. The limited number of inputs needed also demonstrates the feasibility of widely deploying such an approach, saving millions of sleepless patient-nights. We anticipate this directly improving patient outcomes and satisfaction; boosting health provider efficacy by reducing fatigue; and enabling significant cost savings for health systems. Due to the large-scale data set, our model is robust to different healthcare sites, patient demographics, and conditions.

VS assessments are the second most common cause of sleep disruption in the hospitalized patient^16^, and safely reducing these interventions overnight can improve patient sleep quality and quantity while leading to better health outcomes. Multiple studies have measured the quality and quantity of sleep in hospitalized patients around the world, showing significant reductions in sleep quality for patients in North America^1,2,7^, Europe^17^, and Australia^18^, among others. This sleep quality reduction has been linked to a range of adverse health outcomes, including delirium; cardiac and metabolic derangements; cognitive impairment, especially in older adults^4,19^; development of various pain conditions; elevation of inflammatory markers; a decline in self-reported physical health-status^20^; weakened immunity; hypertension; elevated stress hormones; and increased mortality^3^. Importantly, many of these adverse effects are known to lengthen hospitalizations, increasing health utilization and cost without corresponding benefit. Finally, sleep disruption can have an effect on the actual values of the overnight VS—causing erroneous risk alerts and unnecessary interventions—as both blood pressure^21^ and HR^22^ are directly elevated by sleep disturbances.

The benefits of reducing overnight VS monitoring extend beyond patient sleep. Nurses spend between 20 and -35% of their time documenting VS^23^, and roughly 3 min per patient collecting them^24^, accounting for ~10% of their shift with an eight patient census (average of 1.5 VS per patient per night). As healthcare systems seek to maximize efficiency and reduce waste, lean staffing models often hamper compliance with monitoring protocols as clinician capacity is exceeded, leading to delayed or incomplete care^25^, particularly during periods of high acuity or census. Indeed, low nurse-to-patient ratios during overnight shifts have been closely linked with low protocol compliance^26^. Nurses have already adopted strategies to cope with staff and time constraints by triaging patients to different VS measurement frequencies^27^ based upon “gut feeling, sixth sense, or clinical intuition”^25,26,28^. As our algorithm would enable safely and confidently forgoing half of overnight VS measurements, it would result in up to 20–25% workload reduction of an overnight shift, facilitating focus on more acutely ill patients. Such an approach might also align staffing capacity and demand, positively impacting provider stress and reducing burnout, while improving operational efficiency.

Previous approaches to improve patient sleep and satisfaction have been successfully implemented in clinical studies. In the SIESTA research study^10^, laboratory order times were changed to reduce sleep disruptions and clinicians were discouraged from disrupting patient sleep and nudged to consider whether VS assessment was necessary throughout the night for each patient. While the SIESTA-enhanced hospital unit registered significantly fewer entries to patient rooms and corresponding patient sleep disruptions, the protocol leaves the considerable burden of overnight patient stability prediction solely on the clinician and does not assist their decision. A recent randomized controlled trial leveraging an evening risk assessment using the electronic Cardiac Arrest Risk Triage (eCART) score^29^ to facilitate sleep proved the feasibility of this approach^15^. The eCART calculation, however, is complex, incorporating 33 variables including VS and lab results, thus making missing or sparse patient data a problem for the calculation. Similar to MEWS, eCART ignores patient trends, which have been shown to increase the accuracy of clinical deterioration detection^30^, and considers only the latest cross-sectional instance of their values. Our approach to prediction, being fully automated and requiring only a small set of longitudinal VS, can be combined with the laboratory order schedule adjustments and nursing education introduced in SIESTA to achieve higher rates of quiet patient-nights.

While the number of misclassified as stable patient-nights of our algorithm is kept at a rate of 0.02% or 2/10,000, it could potentially deter some risk-averse providers from using it. While we were unable to identify a prior VS trend in those misclassified as stable patient-nights (see Fig. 4), their overnight physiological states are significantly different than those of the correctly classified stable cases (see Fig. 5): as RR and HR, in particular, are considerably higher for the former, we postulate that a simple visual inspection of the sleeping patients during typical nurse rounds should suffice in detecting these misclassified patients, a procedure that is already part of standard nursing guidelines. Based on these significant overnight VS differences, we further propose that real-time HR monitoring could potentially augment our method to identify the misclassified as stable cases (see Fig. 6), similar to their use in improving sleep quality by identifying obstructive apneic episodes^31,32^. Determining which patients should receive overnight continuous monitoring devices remains an open research question.

Predictive machine learning approaches have enjoyed limited clinical adaptation^33^, due to the additional effort they require from doctors and nurses, usually in the form of supplementary data collection or increased workload from health providers. Nevertheless, our solution relies on already existing data pipelines, and intends to reduce the workload of nurses by eliminating unnecessary VS measurements overnight. It does not require any additional data to be collected by providers, leveraging VS captured in the standard workflow.

Our study has several limitations. Although we leveraged a pre-existing health-system risk score and escalation pathway to define instability or deterioration, we did not examine outcomes such as mortality or ICU transfer. This model has not yet been implemented in clinical use, and its acceptance and impact on processes of care are not yet tested. The next version of the model will examine additional endpoints and a pilot implementation is planned to assess feasibility and acceptance on inpatient nursing units. In a production environment, overnight stability predictions will be made at the start of the nursing night shift and predictions will be incorporated into the workflow by integration into nursing tools already in use. Although we emulated a prospective rollout by splitting the data set chronologically, we plan on collecting further surrogate or intermediate outcomes on mortality rates, sleep quality, and patient satisfaction in the course of the deployment of the operationalized version of our production algorithm.

Leveraging routine VSs collected in a large health system, we effectively developed and validated a parsimonious and generalizable deep-learning predictive model to identify patients who are sufficiently stable to avoid overnight sleep interruptions. This model can potentially facilitate the sleep of up to 50% of hospitalized patients, with extremely low risk (2 in 10,000 patient-nights) of classification errors. Once implemented, this model will improve patient healing and experience while lessening the clinical staff burden.

## Methods

### Data description and preprocessing

We used data from the enterprise inpatient electronic health record (EHR; Sunrise Clinical Manager, Allscripts, Chicago, IL) of a large health system in New York, which covers 14 of the health system’s 23 hospitals. All analyses were performed using the Python programming language, version 3.7. This study, including a waiver of consent, was reviewed by the Northwell Health Institutional Review Board and was determined as exempt from International Review Board approval due to the use of de-identified data and the retrospective analysis nature of the data. The study also adhered to the transparent reporting of a multivariable prediction model for individual prognosis or diagnosis (TRIPOD) statement^34^.

The complete de-identified retrospective and prospective data set contains 2,318,506 inpatient hospital admissions and 26,201,030 records of VS between 2012 and 2019 (Table 1). The retrospective data set included all data through April 2019, while the prospective collection ranged from May to August 2019. Patients in our data set were aged between 14 and 89 years (patients older than 89 years were recorded as 89 to protect confidentiality) and were excluded if they were on an obstetrics service. As patients in the ICU are too unstable to consider forgoing overnight VS, only non-ICU VS were used for prediction.

We developed a model that takes a sequence of prior VS for a given patient and predicts the chance of their stability for any given night, in order to determine the necessity of assessing overnight VS. Each record includes HR (b.p.m.), RR (b.p.m.), SBP (mmHg), and Tmpr (°C) measurements, in addition to the corresponding MEWS for early deterioration^13^. This specific risk score, calculated with a simple formula from measured VS (Supplementary Table 1), is a variant of other known and used risk scores, such as EWS and NEWS^35,36^. Two of the MEWS subcomponents (a neurologic assessment [AVPU] and body mass index) had a significant amount of missing data (>80%) and were not included as unique inputs to the model.

An elevated MEWS score indicates risk for clinical instability, including death or need for ICU admission^13^. In 2012, our health system created a custom modification, which was incorporated into the EHR with automatic calculation and display via Arden Syntax Medical Logic Modules^37^. Based on local health-system guidelines, any score ≥7 (score range 0–15) requires intervention per defined protocol (Supplementary Table 2). We defined overnight between the hours of 10 p.m. and 6 a.m., and based upon health-system guidelines, we used an overnight MEWS ≥ 7 to indicate a patient at risk for deterioration.

Unrealistic, possibly mistyped VS values (HR <10 and >300, RR <5 and >47, BP <50 and >300, and Tmpr <20 and >50) were removed. Missing values were imputed using the last observation carried forward method, provided that an earlier measurement was available within a 12-h time window. Records with any missing data after imputation were removed. Additional derived features included the elapsed time and change in risk score since the last recording, hour of the day, and the variance of risk subscores. VS and age variables were scaled to a range between 0 and 1, while time-related features were one-hot encoded.

Each patient visit was comprised of sequences of VS measurements with a minimum length of 8 and a maximum of 42 values (Fig. 1). Any gap in measurements of ≥24 h resulted in the truncation of the sequence and creation of a new sequence from VS measurements after the gap. Each night’s label was represented by a binary variable derived from the highest risk score of the 8-h period following the corresponding patient history; if any score ≥7, the label was positive (1), and negative (0) otherwise.

We split the retrospective data into training (70%), validation (15%), and test (15%) sets by patient visit, so the sequences drawn from one visit were only included in one of the groups (Fig. 2a). While the prediction period was restricted to the night hours in the validation and test sets, we relaxed this restriction in the training set for data augmentation purposes. The prospective data set evaluation emulated an authentic prospective deployment, whereby the model parameters were fixed and predictions were made in a strict chronological order. We further discarded VS for the nights that the algorithm determined as stable, effectively concealing them from the model and avoiding their usage in predictions for subsequent nights.

### Model architecture and training

Our deep-learning model takes the bundle of features sequentially and performs the prediction at the end of the sequence. The model consists of two fully connected layers and five recurrent LSTM memory cells, each with a size of 32, in addition to a fully connected output layer (Fig. 2b). Simple RNNs and bidirectional architectures^38^ were investigated as well, but we achieved better performance and orders of magnitudes faster training time using the cuDNN implementation of LSTM^39^. We found that incorporating dropout^40^ and batch normalization^41^ layers stabilized model performance, resulting in more robust generalization to the test sets. We predicted the actual risk score in a probabilistic programming setting, which gave us a certainty estimate aside from the point estimate; however, we did not find any benefit in our use case of patient stability prediction.

Class imbalance was handled by first randomly removing samples from the negative ones, majority class, until it constituted only 92% of the training set instead of the original 96%. Moreover, the error in predicting positive outcomes was weighted eight times higher in the loss of function compared to the negative ones. Models were implemented in Tensorflow v2.1^42^, and trained on batches of sequences, pre-padded to a length of 42 and with a batch size of 512.

We also evaluated a linear logistic regression model that receives the same input variables as the proposed RNN, using the latest instance of VS measured right before the predicted patient-night rather than a sequence of VS.

### Evaluation

Evaluation was performed in both retrospective test (894,138 nights) and prospective (175,618 nights) settings (see Fig. 2a). Prospective data included a separate 4-month period following the retrospective recordings.

To assess the efficacy of the model, we constructed ROC curves and calculated the area under the ROC curves for both retrospective and prospective datasets using multiple thresholds. True positives (TPs) correspond to accurate prediction of overnight stability (left sleeping and remained stable) and false positives (FPs) to predicted overnight stability (left sleeping) but potentially unstable (MEWS ≥ 7).

As the model provides a probability measure of patient stability, a threshold was required to deduce the final, binary decision (wake or sleep). In order to define the threshold in a clinically relevant manner, we anchored it to the number of FP predictions (let sleep, but potentially unstable) per 10,000 patient-nights. Due to the nature of our model predictions, and in consultation with clinical collaborators, we favored a low FP rate, hence a conservative model. We delineated the clinically applicable region to contain confidence thresholds resulting in missed detections (patients sleeping that are at risk of deterioration) at a maximum of 2 out of 10,000 patient-nights. The threshold used in the prospective testing was derived from the performance of the model on the retrospective test set, with the threshold fixed on the edge of the clinically applicable region.

In addition to the standard ROC curve, a more clinically relevant variant was designed, juxtaposing the number of TP and FP normalized by 10,000 patient-nights, that is, TPclin = TP/(*P* + *N*) and FPclin = FP/(*P* + *N*), where TPclin and FPclin are the clinically relevant TPs and FPs, respectively; TPs and FPs are the previously mentioned TPs and FPs, and *P* represents the positive and *N* the negative cases.

### Reporting summary

Further information on research design is available in the Nature Research Reporting Summary linked to this article.

## Supplementary information

Supplementary Information

Reporting Summary

## Data Availability

The datasets analyzed and generated during the current study are available from the corresponding author on reasonable request.

## Code availability

The implementation of the model and the source code for the algorithm is available from the corresponding author on reasonable request.
