# Supplementary material for: Let Sleeping Patients Lie, avoiding unnecessary overnight vitals monitoring using a clinically based deep-learning model
Source: NPJ Digit Med. 2020 Nov 13;3:149. doi: 10.1038/s41746-020-00355-7 (PMC7666176; doi:10.1038/s41746-020-00355-7)
Supplement: Supplementary file 1 — Supplementary Information [file 41746_2020_355_MOESM1_ESM.pdf]

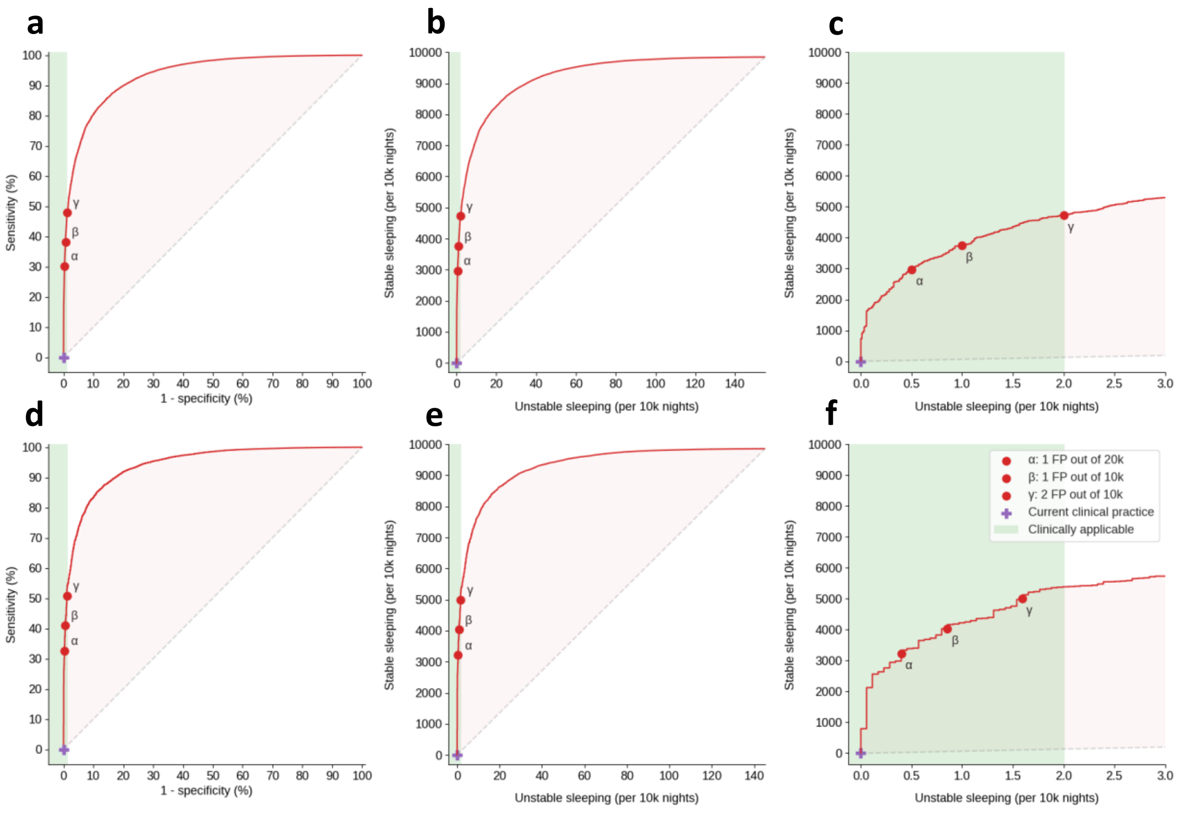

Supplementary Figure 1. Model performance of the logistic regression model illustrated by receiver operating characteristic (ROC) curves and clinically renormalized variants. Panels show the same metrics as in Figure 3.

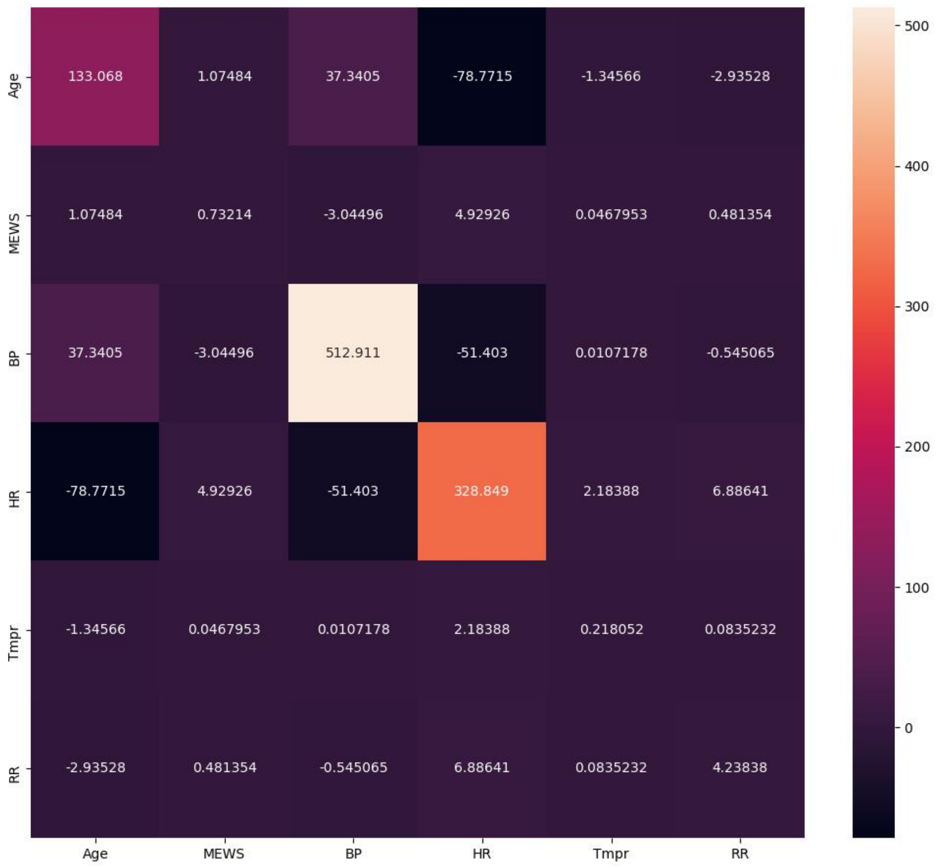

Supplementary Figure 2. Covariance matrix of input variables.

Supplementary Table 1. Modified Early Warning Score (MEWS) calculation, based upon vital sign measurements. Each item is given a score, and the final score is tallied (range 0-15), with higher values indicating greater risk of clinical decompensation.

	**3**	**2**	**1**	**0**	**1**	**2**	**3**
**Systolic Blood Pressure (mmHg)**	<70	70-80	81-100	101-199		≥200	
**Heart Rate (bpm)**		<40	41-50	51-100	101-110	110-129	≥130
**Respiratory Rate (bpm)**		<9		9 - 14	15 - 20	21 - 29	≥30
**Temperature**		<35		35-38·4		≥38·5	
**Neurological Status (AVPU)**				Alert	React Voice	React Pain	Unresponsive
**BMI (kg/m2)***			<18·5		25·1-34·9	≥35	
**Age (y)***					65-74	75-84	≥85

Supplementary Table 2. Health system protocol depending on Modified Early Warning Score (MEWS) value, as incorporated into the electronic health record.

	Health system protocol recommended action
**MEWS 7**	Increase vital sign frequency to every 2 hours
**MEWS 8**	Increase vital sign frequency to every 2 hours<br>+ licensed independent provider evaluation
**MEWS 9**	Increase vital sign frequency to every 2 hours<br>+ licensed independent provider evaluation<br>+ consider rapid response team evaluation
**MEWS 10+**	Increase vital sign frequency to every 2 hours<br>+ licensed independent provider evaluation<br>+ consider rapid response team evaluation<br>+ change in level of service
